# Prof. Sim

**Published:** 1894-07

**Authors:** 


PROF. SIM, of Memphis, read before a meeting of doctors and lawyers in Memphis recently, a paper on "Asexualization as a means of arresting the propagation of criminals" and it was discussed by able members of both professions. It was very much in line with the views expressed by Dr. Daniel in his paper before the International Medico-Legal Congress at Chicago last summer, which attracted so much attention.

Agitation of this subject,—the substitution of castration for capital punishment for certain crimes—has now become a movement,—the popular sentiment in favor of abolition of capital punishment as useless and cruel—the only remaining remnant of the triple barbarism, slavery, polygamy and hanging—is daily growing, and will soon culminate in Texas in a demand for legislation to effect the change. In Austin the subject has been much discussed, and prominent legal gentlemen will petition Gov. Hogg —(who by the bye is a strong advocate of the change), to recommend it in his message to the legislature; and the representatives from this (Travis) county will be asked to draw up a bill embodying the suggestions put forward in Dr. Daniel's paper and recently by Prof. Sim. We are gratified to see a similar movement in Tennessee originated by our able and distinguished colleague, Prof. Sim, and hope to see it become general throughout the United States.

